# Prevalence of Neurobehavioral, Social, and Emotional Dysfunction in Patients Treated for Childhood Craniopharyngioma: A Systematic Literature Review

**DOI:** 10.1371/journal.pone.0076562

**Published:** 2013-11-05

**Authors:** Gabriel Zada, Natalie Kintz, Mario Pulido, Lilyana Amezcua

**Affiliations:** 1 Department of Neurosurgery, Keck School of Medicine of USC, Los Angeles, California, United States of America; 2 The George and MaryLou Boone Center for Parkinson’s Disease Research, Keck School of Medicine of USC, Los Angeles, California, United States of America; 3 Department of Neurology, Keck School of Medicine of USC, Los Angeles, California, United States of America; 4 Department of Biochemistry and Molecular Biology, Keck School of Medicine of USC, Los Angeles, California, United States of America; University of Virginia Health Science Center, United States of America

## Abstract

**Background:**

Craniopharyngiomas (CP) are locally invasive and frequently recurring neoplasms often resulting in neurological and endocrinological dysfunction in children. In addition, social-behavioral impairment is commonly reported following treatment for childhood CP, yet remains to be fully understood. The authors aimed to further characterize the prevalence of neurobehavioral, social, and emotional dysfunction in survivors of childhood craniopharyngiomas.

**Materials and Methods:**

A systematic literature review was conducted in PubMed to identify studies formally assessing neurobehavioral, social, and emotional outcomes in patients treated for CP prior to 18 years of age. Studies published between the years 1990-2012 that reported the primary outcome (prevalence of neurobehavioral, social, emotional/affective dysfunction, and/or impaired quality of life (QoL)) in ≥10 patients were included.

**Results:**

Of the 471 studies screened, 11 met inclusion criteria. Overall neurobehavioral dysfunction was reported in 51 of 90 patients (57%) with available data. Social impairment (i.e. withdrawal, internalizing behavior) was reported in 91 of 222 cases (41%). School dysfunction was reported in 48 of 136 patients (35%). Emotional/affective dysfunction was reported in 58 of 146 patients (40%), primarily consisting of depressive symptoms. Health related quality of life was affected in 49 of 95 patients (52%). Common descriptors of behavior in affected children included irritability, impulsivity, aggressiveness, and emotional outbursts.

**Conclusions:**

Neurobehavioral, social, and emotional impairment is highly prevalent in survivors of childhood craniopharyngioma, and often affects quality of life. Thorough neurobehavioral/emotional screening and appropriate counseling is recommended in this population. Additional research is warranted to identify risk factors and treatment strategies for these disorders.

## Introduction

Craniopharyngiomas (CP) are neoplasms that typically develop in the sellar and/or suprasellar region, often resulting in a significant degree of neurological and/or endocrinological morbidity in children, including visual loss, panhypopituitarism, and diabetes insipidus [1,2]. Although they are not malignant tumors, local tumor invasion into critical neurovascular structures including the hypothalamus, frontal lobe, ventricles, cranial nerves, and circle of Willis often makes complete tumor resection unfeasible and unsafe, thereby resulting in subtotal tumor resection and frequent recurrence and/or progression[3]. Standard treatment regimens for CP have therefore recently evolved towards maximal safe tumor resection with administration of adjunctive radiation therapy for residual tumor, with an emphasis on preservation of neurological and endocrinological function[2,4,5]. Despite multimodal treatment strategies for patients with CP, however, the propensity for tumor recurrence and future disability remains extremely high[2]. 

In recent years, several studies have assessed the long-term social impact of hypothalamic obesity and other comorbidities associated with childhood craniopharyngioma and iatrogenic treatment effects[6–9]. In addition, aside from the neurocognitive and endocrinological morbidity associated with childhood CP, many patients suffer from challenging neurobehavioral, social, and emotional issues. The tremendous impact caused by neurobehavioral dysfunction and affected quality of life often poses a formidable challenge for patients treated for CP, their families, and educational workers. For instance, emotional irritability, anger outbursts, social isolation, anxiety, and depression are common descriptors of behavior in children with craniopharyngiomas. These effects have the propensity to permeate many aspects of daily life and negatively influence the ability for survivors of childhood CP to perform in school or the workplace, and form functional relationships. The reasons for neurobehavioral and social dysfunction in children with CP are likely multifactorial, and are potentially associated with numerous patient and treatment parameters, including tumor location/size, degree of hypothalamic involvement, hypopituitarism, obesity, presence of hydrocephalus, surgical interventions and approaches, and radiation history, among several others.

The degree to which neurobehavioral, social and emotional dysfunction prevails in patients treated for childhood CP, and subsequently affects health-related QoL, is limited primarily to case series and isolated case reports, and remains to be further explored. Improved understanding of the prevalence of these impairments, and any potential risk factors associated with their development, may help identify patients that are most likely to develop neurobehavioral dysfunction and improve early screening, intervention, and treatment for these conditions. In order to more accurately characterize the prevalence and burden of neurobehavioral, social, and emotional dysfunction in this population, a systematic literature review was conducted to identify studies formally assessing neurobehavioral function in patients with a history of treated childhood craniopharyngioma. In particular, this review focused on social-behavioral and emotional outcomes rather than neuro-cognitive function.

## Materials and Methods

### Ethics Statement

This study was conducted according to the Helsinki human subject doctrine and approved by The USC Ethics Committee/Institutional Review Board.

### Preliminary Search Strategy

**Figure 1:**
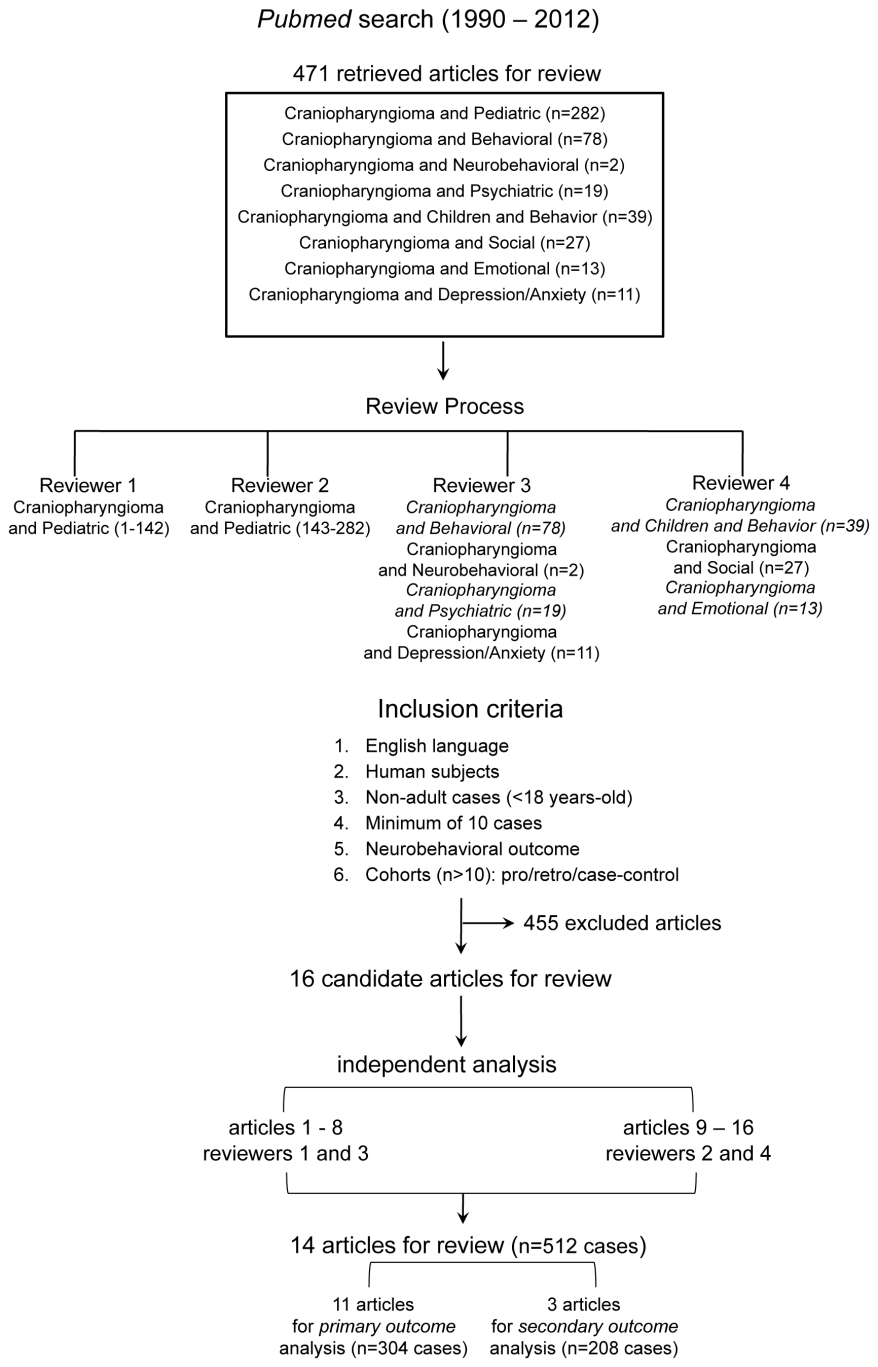
Flow diagram detailing the flow of information and study selection through the systematic review process.

The primary objective of the current search strategy was to identify all published studies reporting the incidence of neurobehavioral, social, or affective/emotional outcomes in patients diagnosed and treated for childhood craniopharyngioma (≤18 years of age). A detailed systematic search strategy was developed which included all four authors and was conducted using the PubMed database. All search results were limited to studies published between 1990-2012 in order to preferentially select for patients treated with current multimodality regimens and screened using current neuropsychological assessments. Search criteria were based on keywords related to CP in children and neurobehavioral, social, and emotional outcomes (Figure 1) using the following search terms: “Craniopharyngioma and pediatric,” “craniopharyngioma and behavioral,” “craniopharyngioma and neurobehavioral,” “craniopharyngioma and psychiatric,” “craniopharyngioma and children and behavior,” “craniopharyngioma and social,” "craniopharyngioma and emotional," and “craniopharyngioma and depression/anxiety.” In addition, the reference lists of all relevant articles were examined for reports of additionally relevant studies. The study was conducted in accordance with the 2009 preferred reporting items for systematic reviews and meta-analyses (PRISMA) guidelines for methodology of systematic reviews[10]. 

### Inclusion and Exclusion Criteria

For inclusion in the review, studies had to: 1) Be written in the English language, 2) Involve human subjects only, 3) Include non-adult cases, (defined as age ≤18) at time of craniopharyngioma diagnosis and treatment, 4) Include a minimum number of 10 cases reported in the series (in order to maximize homogeneity of screening assessments used), and 5) Conduct formal assessment of at least one neurobehavioral/social/emotional outcome using a validated test. Cohort studies, case-control studies, and case-series including greater than 10 subjects were included, whereas case reports were excluded. Posttest data after any treatment of CP, including surgical resection and/or radiation, was sufficient for inclusion. Studies that included a cohort of individuals treated for CP as children as part of a larger study were also included if the analysis for this specific sub-cohort was reported separately. Studies were excluded if: 1) They focused on a clinical intervention, 2) Analysis did not separate data from age (≤18 versus >18 years old) at diagnosis, 3) Analysis did not separate CP from other study or tumor groups. 

### Implementation of search strategy and study selection process

A preliminary review of the search results was conducted. Four reviewers were assigned a portion of the search results (Figure *1*), and independently screened the titles and abstracts of all pertinent results, applying the inclusion/exclusion criteria. If it was unclear from the abstract whether a study met the entry criteria, the full text was assessed independently. A secondary review was subsequently conducted in which full text articles of the studies included from the preliminary search were retrieved. Two reviewers independently assessed the eligibility of each of the full text articles based on the inclusion/exclusion criteria. Following the independent review, two reviewers jointly determined which articles would be included. A total of 4 discrepancies were noted and resolved through following review by a third or fourth independent reviewer. All excluded studies and reasons for exclusion were documented. An electronic database was developed, and two sets of two reviewers were assigned to half the studies for formal in-depth review. 

### Information extraction and data collection process

For included studies, information was collected regarding characteristics including year of study, study sample size, study design, modalities of craniopharyngioma treatment (i.e. surgical, radiation, or both), age at treatment, follow-up times, neuropsychological assessments used, sex of patients, and proportions of patients with hypopituitarism, diabetes insipidus, hypothalamic involvement, obesity, or hydrocephalus. 

The primary outcome(s) recorded were: Prevalence of overall neuro-behavioral dysfunction, social impairment, school dysfunction, emotional/affective dysfunction, and prevalence of patients with impaired health-related quality of life. Secondary outcomes analyzed (if primary outcomes were not available) included reports of mean or median cohort scores of neurobehavioral, social, emotional, or QoL assessments, and any control group scores recorded. For calculation of combined prevalence, a weighted proportion was calculated from all studies reporting pertinent results, based on the sample size with available data.

### Potential for Bias

 In order to limit potential bias, inclusion and exclusion criteria were defined prior to conducting the literature search. All studies included in this systematic review were reviewed by two independent reviewers, and extracted data from the two independent reviewers was compared to ensure accuracy. Discrepancies were resolved through review and discussion with all authors until consensus was achieved. 

There is a considerable amount of variation across the different studies with respect to questionnaires and techniques used to measure neuropsychological, social, school and emotional/affective disturbances. Only studies that used validated assessment tools were included. Assessments used to analyze neurobehavioral dysfunction included: Child Behavior Checklist (CBCL) total score, Youth Self Report total score, Behavior Rating Inventory of Executive Function (BRIEF), Vineland Behavior Adaptive Composite, and the Children’s Global Assessment Scale (C-GAS). Social function was assessed using: CBCL, YSR, Personality Inventory Scale for Children (PIC), Kidscreen-52, and semi-structured interviews with patients and/or parents. School performance was assessed via interviews with parents and/or teachers, and the School Achievement Test. Emotional/affective disturbances were assessed using the Beck Depression Inventory, CBCL, YSR, Personality Inventory Scale for Children, Health Utilities Index Mark 2, Short Mood and Feelings Questionnaire (SMFQ), and the Multistory Depression Inventory for Children (MDI-C). Studies administering the questionnaire directly to patients, or to parents as proxy respondents, were included. Information attainment was also quite variable, in that questionnaires administered over the phone, via mail, or in person were included. 

### Quality assessment

The formal quality assessment described above ensured that well-validated neurocognitive tools were used in all studies included. As the vast majority of studies had a cohort or case-control design, and therefore lacked randomization, a quality review of randomization was not performed.

## Results

### Preliminary and Secondary Search Results

 Of the 471 studies identified in the preliminary search, 11 met inclusion criteria for analysis of the primary outcome (prevalence of neuro-behavioral, social, or emotional impairment), and 3 additional articles reported mean values only of the case population without prevalence rates. (Table *1*) Formal neuropsychiatric assessments were utilized in all included studies and included, but were not limited to, the following: CBCL, Personality Inventory Scale in Children, Pediatric Quality of Life Inventory (Persil), Beck Depression Inventory, HU-12, Kid screen 52, Multistory Depression Inventory for Children (MDI-C), World Health Organization Quality of Life (WHO-QOL), Health Utilities Index (HUI), Psychological General Well-being (PGWB), Short Mood and Feelings Questionnaire (SMFQ), Young Adult Checklist, and Child Health Questionnaire (CHQ-PF50). 

**Table 1:** General characteristics of the included studies.

**Author**	**Year**	**Setting**	**Study Design**	**Sample Size (n)**	**Patients Available for Analysis, (n)**	**Percent Female (%)**	**Age Range (years)**	**Mean follow-up time (years)**
***Primary Outcome (Prevalence)***								
Anderson A	1997	Single Center, Hospital	Cohort	24	20	45	3-17	3.2
Riva D	1998	Single Center, Hospital	Cohort	22	12	50	5-14	10
Merchant T	2002	Single Center, Hospital	Cohort	30	30	57	1-15	6.1
Poretti A	2004	Single Center, Hospital	Cohort	25	21	42	2-10	11.3
Pierre-Kahn A	2005	Single Center, Hospital	Cohort	14	14	n/a	4-13	2
Waber D	2006	Single Center, Hospital	Cohort	10	10	n/a	3-15	9
Pedreira C	2006	Single Center, Hospital	Case-Control	46	12	42	1-11	6.4
Puget S	2007	Single Center, Hospital	Cohort	88	66	57	1-16	7
Ondruch A	2011	Single Center, Hospital	Cross-Sectional	43	27	57	2-16	n/a
Laffond C	2012	Single Center, Hospital	Cohort	29	27	44	7-24	4
Gautier A	2012	Two Centers, Hospital	Cohort	171	65	n/a	4-15	16.8, 17.3
***SecondaryOutcome(Groupmeans***)								
Sands S	2005	Single Center, Hospital	Cohort	29	29	41	1-18	6
Kendall-Taylor	2005	Multi-Center, Clinics	Cohort	393	152	46	0-18	n/a
Dolson EP	2009	Single Center, Hospital	Cohort	27	27	59	3-17	3

### Patient Demographics

 A total of 502 patients were included among all 11 studies contributing to the primary outcome, with available prevalence rates specifically reported in 304 patients (Table *1*)[4,11–20]. Mean follow-up times from treatment ranged from 2 to 17 years. A majority of studies were cohort studies based from single center hospitals. Three additional studies reported secondary outcomes (group means) in 208 patients of a total 451 patients[21–23]. Interventions and comorbidities of the sample populations are reported in Table *2* *.*

**Table 2:** Interventions and co-morbidities of the sample population.

**Author**	**Sample Size (n)**	**Surgical Intervention (n)**	**XRT (%)**	**Recurrence (%)**	**HPS (%)**	**DI (%)**	**HCP (%)**	**Obesity (%)**	**HTS (%)**	**Neuropsychiatric Tool/ Assessment Utilized**
Anderson A	24	20	85	35	85	n/a	30	n/a	n/a	CBCL
Riva D	22	12	0	33	75	n/a	0	58	n/a	PIC
Merchant T	30	30	77	30	100	55	23	n/a	n/a	HUI
Poretti A	25	25	12	25	95	92ⱡ	48ⱡ	67ⱡ	81ⱡ	PedsQL, CBCL, FMH, Youth Self Report
Pierre-Kahn A	14	14	n/a	n/a	n/a	n/a	n/a	64	50	Global Assessment Scale
Waber A	10	10	70	n/a	n/a	n/a	n/a	n/a	n/a	Beck Depression Inventory
Pedreira C	46	18	50	72	89	78	n/a	50	n/a	PGWB, Children's Manifest Anxiety Scale. Short Mood Feeling Questionnaire
Puget S	88	66	35	36	n/a	9ⱡ	53	30	27	HU-12 (funtional outcome scale)
Ondruch A	43	27	15	15	n/a	22	n/a	n/a	n/a	CBCL
Laffond C	29	14	100	14	97	n/a	34	59	79	QoL (Kidscreen 52), MDI-C, and proxy-reports of QoL
Gautier A	171	33, 32	24, 30	44	n/a	14§, 14ⱡ	n/a	75ⱡⱡ, 55ⱡ	n/a	WHO-QoL
***SecondaryOutcome(Groupmeans***)										
Sanda S	29	29	n/a	21	n/a	n/a	45	n/a	n/a	CBCL
Kendall-Taylor	373	138	44	23	100	61	n/a	n/a	n/a	QoL-HGHDA, NHP, patient life situation form
Dolson EP	27	27	100	n/a	85	63	67	48	n/a	CBCL

ⱡ Reference group: number available Table 1, § Out of 29, ⱡⱡ Out of 28, HPS-hypopituitary syndrome, DI-diabetes insipidus,

HCP-hydrocephalus, HTS-hypothalamic syndromeNeurobehavioral, Social, and School Dysfunction in patients treated for Childhood Craniopharyngioma

 Primary outcomes from the eleven included studies are reported in Table *3*. Overall neurobehavioral dysfunction was reported in 51 of 90 children (57%) with available data (weighted mean=56.7%)[11,12,15–17]. Social impairment was specifically reported in 91 of 222 (41%) children treated for craniopharyngiomas (weighted mean=41.7%) [4,11,12,14,16–18,20]. Social impairment consisted primarily of behaviors such as withdrawal, internalizing, and hostility. School dysfunction was specifically reported in 48 of 136 patients (35%, weighted mean= 35.3%)[4,11,12,16–18]. Difficulty in maintaining relationships was reported in 17 of 43 patients (40%)[12,17]. 

**Table 3:** Proportions and cumulative weighted averages of neurobehavioral, social, school, and emotional/affective dysfunction derived from 11 studies with relevant data.

		**Result N (%)**			
**Author**	**Year**	**Neurobehavioral Dysfunction**	**Social Impairment**	**School Disturbances**	**Emotional/Affective Dysfunction**
Anderson CA	1997	13/19 (68)	14/19 (74)	13/18 (72)	-
Riva D	1998	-	11/12 (92)	3/12 (25)	12/12 (100)
Merchant TE	2002	-	-	-	12/29 (41)
Poretti A	2004	13/24 (54)	11/24 (46)	10/20 (50)	4/24 (17)
Pierre-Kahn	2005	11/14 (79)	5/14 (36)	4/14 (29)	9/12 (75)
Waber DP	2006	--	-	-	5/10 (50)
Pedreira	2006	9/12 (75)	-	-	2/10 (20)
Puget S	2007	-	5/45 (11)	9/43 (21)	-
Ondruch A	2011	-	8/27 (30)	--	3/27 (11)
Laffond C	2012	5/21 (24)	11/22 (50)	9/29 (31)	11/22 (50)
Gautier A	2012	-	26/59 (44)	-	-
**TOTAL**		51/90 (57)	91/222 (41)	48/136 (35)	58/146 (40)
**Weighted Averages**		56.67%	41.72%	35.30%	39.73%

One additional study reported mean values for a cohort of 27 patients treated with radiation and surgery for childhood CP[21]. Although baseline CBCL values were not abnormal for the group as a whole, the authors noted that worsened neurobehavioral outcomes were correlated with time from treatment, obesity, presence of diabetes insipidus, and presence of hydrocephalus.

### Emotional/Affective Dysfunction in patients treated for Childhood Craniopharyngioma

Emotional-affective dysfunction was reported in 58 of 146 patients (40%) with available data (Table *3*), and frequently included depression, anxiety, mood swings, irritability, and emotional outbursts (weighted mean=39.7%)[12–19]. Depressive symptoms were specifically reported in all 58 patients with emotional dysfunction, and anxiety in 8 of 12 patients with pertinent data [15]. 

### Quality of Life in Survivors of Childhood Craniopharyngioma

Quality of life was screened and reported impairment as a prevalence in 95 patients, which was reduced in 49 patients (52%)[4,13,17]. QoL was most often affected due to social and body image issues rather than neurological or cognitive impairments. Although they did not report prevalence of reduced quality of life, 2 additional studies reported affected QoL in cohorts of patients with treated craniopharyngiomas, particularly in social functioning[22,23]. Although Sands et al did not report affected mean values for 22 CP survivors in overall psychosocial quality of life, low average responses were reported for “social functioning” and “parent impact of emotion”[23]. The authors cited “social problems” and “somatic problems” as the most profoundly affected clinical categories. Kendall-Taylor et al reported affected quality of life outcomes across all groups, and a correlation between worsened QoL and age of onset of CP and age of growth hormone deficiency[22].

## Discussion

Neurobehavioral, social, and emotional dysfunction were found to be relatively prevalent manifestations in survivors of treated pediatric craniopharyngioma. Our review suggests that between one-third and one-half of patients with treated childhood-onset CP have impairments in social function, school, and maintaining relationships. Forty percent of patients have some degree of emotional/affective dysfunction, with depression comprising a majority of these symptoms. In particular, these social and emotional ramifications have a profound impact on quality of life in over half of patients’ lives. Importantly, many cases appear to be more severely affected in the realms of social, behavioral, and emotional function than they are by neuro-cognitive or physical impairments[11,14,16–18,24]. For instance, some studies focusing primarily on neurocognitive performance in survivors of treated childhood CP have reported normal IQ scores in addition to normal achievement, attention, and verbal memory[14,19,25,26]. Rather, neurocognitive impairments were associated with depression, somatic concerns, and deficits in executive function and spatial working memory[18,19]. Social and emotional dysfunction in pediatric patients with CP may have a severe impact on mood, attention, familial and other interpersonal dynamics, and school function. Common social manifestations arising in patients with childhood CP include internalizing behavior, withdrawal, and social isolation[8,25,27]. Additional behaviors including emotional outbursts, impulsivity, and aggressiveness have also been frequently described in this population[11,14,18,28].

 The current review adds to the body of literature defining impaired social-behavioral and emotional dysfunction in survivors of childhood craniopharyngioma, and underscores the need for thorough long-term screening and counseling in this patient population. According to a study by Anderson et al, only 3 of 20 children (15%) with treated CP had normal social behavior and school function[11]. In a study by Ondruch et al, the authors determined that a much higher prevalence of social-behavioral dysfunction existed in CP patients than did physical or cognitive dysfunction[14]. In particular, a high proportion of depression, anxiety and withdrawal were noted. The most commonly identified problems identified included inability to control emotions, difficulties in learning, unsatisfactory peer relationships, and unattractive appearance resulting from hormonal disorders[14]. In the series reported by Riva et al, children were especially likely to have inability to withstand frustration and unmotivated fits of anger[18]. In a qualitative article by Jackson, the authors noted that “Some patients became socially isolated…several parents commented that their child became more tentative, angry, or lethargic. In one case, a mother stated that behavioral changes associated with the condition destroyed the formerly positive relationship with her son”[27].

### Etiology of Social and neurobehavioral dysfunction in childhood Craniopharyngioma

The reasons for the high prevalence of social-behavioral and emotional dysfunction in treated craniopharyngioma patients are likely to be multifactorial, and remain to be completely understood. Although outside of the scope of the current systematic review, there is a high prevalence of co-morbid conditions in patients treated for CP, including hypothalamic involvement, hormonal manifestations (hypopituitarism and DI), obesity, and presence of hydrocephalus. Additionally, both neurobehavioral and social dysfunction appears to be disproportionally higher when compared to other treated benign intracranial tumors arising in the pediatric population[29]. Additional potential risk factors which merit consideration with regard to etiology of neurobehavioral disease include age at diagnosis and treatment, time since treatment, tumor size, requirement for CSF shunting, surgical and radiation history, and several others. Neurobehavioral compromise is therefore likely to arise not from one specific etiology, but from a variety of these sources, with particular mechanisms such as frontal lobe dysfunction, hormonal imbalance, and hypothalamic/diencephalic dysfunction each warranting further study. Cranial irradiation following brain tumor resection has also been noted to correlate with neurocognitive changes[30–32]. Finally, many patients and families maintain a chronic fear of tumor recurrence and anxiety regarding prior treatments[27,33].

One intriguing theory behind the impaired social interaction in children with craniopharyngiomas may relate to previously unexplored neuro-endocrinological aspects of the disease, in particular oxytocin and desmopressin deficiency. Considering the known propensity for hypothalamic involvement in children with CP, and the high incidence of permanent diabetes insipid us (loss of functional desmopressin production and secretion) correlating with hypothalamic, infundibular, and neurohypophyseal dysfunction, we postulate that oxytocin deficiency may also be a common development in these patients, and may partially contribute to the observed social dysfunction. Although no data is currently available to support this theory, recent experiments performed following inhaled administration of oxytocin have demonstrated improved potential for trust and social connectivity in children with autism spectrum disorder, conduct disorders, attachment disorders, anxiety, and depression[34,35]. The prosocial effects of oxytocin are known to play an important role in developing attachment and trust, social memory, and fear reduction[36–38]. In an experimental primate study by Smith et al, in which oxytocin or oxytocin-receptor antagonists were administered to marmoset monkeys, the authors demonstrated that oxytocin activity played a role in altering partner-directed social behavior during pair interactions[39]. The authors concluded that central oxytocin may facilitate the process of pair-bond formation and social relationships in marmoset monkeys[39]. The paucity of data regarding oxytocin levels in this population underscore the need for more research in this area; The exact role of oxytocin deficiency in patients with treated childhood CP remains to be further explored.

### Risk Factors Associated with Worsened Neurobehavioral Outcome

Although the nature of our systematic review did not permit conduction of a meta-analysis with regard to identifying risk factors correlating with worsened neurobehavioral and social outcomes, primarily because of the heterogeneity of parameters studied across various studies and small numbers of patients per study, individual studies did report selected risk factors correlating with worsened social-behavioral and emotional outcomes. In a retrospective study by Sands et al examining quality of life, and using the CBCL to measure social and behavioral outcomes in children, the authors noted that factors correlating with worsened outcome included retrochiasmatic tumor location, recurrence, and additional surgery[23]. In a large study measuring quality of life in 152 adults treated for childhood CP, worsened QoL was associated with age of onset and onset of GH deficiency[22]. Another study by Dolson et al reviewed social and behavioral outcomes in 27 children treated for CP using the Achenbach CBCL score. They determined that risk factors for worsened outcomes included diabetes insipid us, CSF shunting, requirement for an Ommaya reservoir (for intermittent cyst drainage), and low peak pre-irradiation Growth Hormone levels[21]. In a study by Pedreira et al assessing health-related quality of life on 25 patients treated for childhood QoL, risk factors correlating with worsened functional neurobehavioral outcomes included hypothalamic involvement, younger age of onset, presence of hydrocephalus, and tumor recurrence. Taken together, these findings further suggest that the degree of hypothalamic involvement, endocrinological impairment, and tumor recurrence are likely to correlate with severity of social-behavioral and emotional impairment.

### Limitations of Current Study

This systematic review assessed the prevalence of neurobehavioral, social, school and emotional/affective dysfunction in patients diagnosed and treated for craniopharyngioma as a child. Data from the studies included in this systematic review consistently demonstrate that there is high prevalence of neurobehavioral, social, school and emotional/affective dysfunction in patients diagnosed and treated with CP as a child. There is, however, a wide range of variability in factors analyzed, including age at diagnosis, degree of hypothalamic involvement and resection, time since treatment, and variations in treatment approach across the different studies included in the review. Furthermore, there is a high prevalence of clinical factors including diabetes insipid us and obesity in study populations analyzed, which may additionally act as confounding variables. Analyses addressing the significant impact of these variables could not be assessed due to the limited amount of data and small sample sizes. 

In addition to limitations from the wide variety in patient characteristics included that may have confounded these findings, the current study is inherently limited by the heterogeneity of neuropsychological assessments utilized and the means of conducting them, thereby potentially introducing informational bias into each study. Potential for selection bias may derive from the recruitment strategy and inclusion criteria of each study (i.e. characteristics of patients with treated CP that did or did not participate in the studies), as well as during the course of follow-up. Finally, the potential for publication bias is an inherent limitation of any systematic review. These limitations highlight the need for prospective studies with larger patient samples using homogenous assessments in order to better understand the influence and potentially confounding effects of these variables.

## Conclusions

 Patients with childhood craniopharyngioma treated by surgical resection and/or radiation demonstrate a high prevalence of long-term neurobehavioral, social, and emotional impairment in addition to somatic concerns. Social impairment in children treated for CP appears to be characterized by withdrawal, internalization, emotional outbursts, aggressiveness, and a high prevalence of anxiety and depression, which may contribute to strained familial relationships and/or school behavior. The degree of social and emotional dysfunction may affect children more severely than neurocognitive deficits, and often results in strained familial relationships and school dysfunction. In comparison with other benign intracranial tumors, CP appears to be associated with a disproportionally high prevalence of social and neurobehavioral issues. Although likely multifactorial, the reasons for these outcomes remain to be fully understood. Co-morbid conditions including hypothalamic and frontal lobe dysfunction, endocrine deficiencies, hydrocephalus, radiation effects, and somatic concerns relating to hypothalamic obesity need to be considered. Further experimental studies may be warranted to formally address whether hypothalamic or infundibular dysfunction resulting in oxytocin deficiency precludes the ability to formulate socially appropriate relationships in children treated for CP. In any event, thorough screening and counseling for neurobehavioral and mood-related disease is warranted following treatment for CP in children.

## Supporting Information

Checklist S1 **PRISMA Checklist.**
(DOC)
